# Patterns of intersectional tumor volumes in T2-weighted MRI and [^18^F]FET PET in adult glioma: a prospective, observational study

**DOI:** 10.1038/s41598-024-73681-5

**Published:** 2024-10-04

**Authors:** Jonathan Weller, Marcus Unterrainer, Markéta Sonderer, Sophie Katzendobler, Adrien Holzgreve, Annamaria Biczok, Patrick N. Harter, Joerg-Christian Tonn, Nathalie L. Albert, Bogdana Suchorska

**Affiliations:** 1 Department of Neurosurgery, LMU University Hospital, LMU Munich, Munich, Germany; 2 Department of Radiology, LMU University Hospital, LMU Munich, Munich, Germany; 3 Department of Nuclear Medicine, LMU University Hospital, LMU Munich, Munich, Germany; 4 Center for Neuropathology and Prion Research, LMU University Hospital, LMU Munich, Munich, Germany; 5 German Consortium for Translational Cancer Research (DKTK), Partner site Munich, Heidelberg, Germany; 6 Department of Neurosurgery, Heidelberg University Hospital, Heidelberg, Germany

**Keywords:** Imaging, Positron emission tomography, Glioma, Glioblastoma, Radiotherapy, Cancer in the nervous system, Cancer imaging

## Abstract

Brain tumor volumes as assessed by magnetic resonance imaging (MRI) do not always spatially overlap with biological tumor volumes (BTV) measured by [^18^F]Fluoroethyltyrosine positron emission tomography ([^18^F]FET PET). We prospectively investigated volumetric patterns based on the extent of tumor volume overlap between the two modalities. Eighty-six patients with newly diagnosed glioma who had undergone MRI and [^18^F]FET PET between 2007 and 2009 were included in this prospective study and (re-)classified according to CNS WHO 2021 (Classification of Tumors of the Central Nervous System by the World Health Organization). Four different patterns of volume overlap were defined mathematically according to the extent of overlap between MRI-based T2 tumor volume (non-enhancing tumor volume, nCEV) and BTVs. Progression-free (PFS) and overall survival (OS) were determined. Seventy patients were diagnosed with isocitrate dehydrogenase wildtype (IDHwt) glioblastoma and 16 with IDH-mutant glioma, respectively. The most common pattern was characterized by a larger non-contrast-enhancing tumor volume (nCEV) that enclosed all or most of the BTV and was observed in 46 patients (54%) (pattern 1). This pattern was more frequent in IDH-mutant gliomas than in IDH-wildtype glioblastoma (81% versus 47%, *p* = 0.02). In multivariate analyses, pattern 1 was associated with prolonged PFS (HR 0.59; 95 CI 0.34-1.0; *p* = 0.05), but not OS (HR 0.66; 95 CI 0.4–1.08; *p* = 0.1). For OS, presence of an IDH mutation (*p* = 0.05) and lower age (*p* = 0.03) were associated with prolonged OS. The spatial relation between nCEV and BTV varies within and between glioma entities. Most frequently, a larger nCEV encases the BTV. Some patients show spatially dissociated nCEVs and BTVs. Not accounting for this phenomenon in surgery or radiotherapy planning might lead to undertreatment.

**Supplementary Information:**

The online version contains supplementary material available at 10.1038/s41598-024-73681-5.

## Introduction

Gliomas are classified based on molecular and histological features as recommended by the World Health Organization (WHO) and the Consortium to Inform Molecular and Practical Approaches to CNS Tumor Taxonomy (cIMPACT)^1–3^. Overall, gliomas occur at an age-adjusted incidence rate of 4–6/100’000 population per year^4^. Standard therapy for adult gliomas comprises tumor resection if safely feasible, radiotherapy, alkylating agent chemotherapy and combinations thereof in high-grade and high-risk lower-grade gliomas^5,6^. Clinical decision making, radiotherapy planning and response assessment in affected patients rely on non-invasive imaging modalities such as magnetic resonance imaging (MRI). In addition to anatomic, MRI-based imaging, amino acid-based positron emission tomography (PET) utilizing O-(2-[18F]fluorethyl)-L-tyrosine ([^18^F]FET PET) provides valuable information on tissue vitality, metabolism and can be useful for prognostication^7–11^. Recommendations on clinical use of PET in gliomas, especially in the context of therapy response assessment, have been published by the Response Assessment in Neuro-Oncology working group (RANO group)^7^. PET-derived biological tumor volumes (BTVs) are often larger than contrast enhancing volume (CEV) portions in primarily enhancing tumors, but smaller than the non-enhancing volume (nCEV) portions^9^. However, morphological MRI-based tumor volumes might not always spatially overlap with BTV^12^. Comprehensive definition of a tumor volume, potentially incorporating different imaging modalities, is essential for therapy planning, especially for radiotherapy. Besides potential clinical implications regarding therapy planning, different volume patterns might inherently be associated with molecular markers or outcome. One methodological approach might be the definition of intersectional tumor volumes defining the pattern of overlap between MRI and PET volumes prior to any therapeutic intervention, e.g., nCEV enclosed in BTV or vice versa: These intersectional volumes have been shown to correlate with grading in anaplastic astrocytomas and glioblastomas^12^. Intersectional tumor volume patterns could be investigated in an even more detailed fashion, e.g., as follows: nCEV larger that the BTV and enclosing all or most of it (I), or conversely a BTV larger than the nCEV and enclosing all or most of it (II), or similar volumes in both modalities with one overlapping the other (III), or lastly similar volumes that show minimal overlap, but large exclusive volumes (IV).

In this context, the aim of our study was to define different patterns mathematically and compare localization and extent of anatomic tumor volumes as assessed by MRI with [^18^F]FET PET-based BTVs in newly diagnosed glioma. We set out to investigate the patterns with regards to clinical and molecular parameters as well as progression-free and overall survival.

## Materials and methods

### Setting and patient inclusion criteria

Patients with newly diagnosed glioma between 2007 and 2009 who underwent histological sampling at the Department of Neurosurgery of the LMU University Hospital Munich and were eligible for MRI and [^18^F]FET PET scans were included in this prospective study that was waived by our institutional ethics committee (NCT01089244; all experimental protocols were approved by the Ethics Committee of the Faculty of Medicine at LMU Munich; local project number: 325 − 11). The study was conducted in accordance with the Declaration of Helsinki and institutional guidelines as well as regulations. Informed written consent to participate in the study was obtained from all patients. Histopathological and molecular analyses were conducted at the Center for Neuropathology at the LMU University Hospital Munich. The CNS WHO 2021 classification was applied to all samples^3^. Informed consent was obtained from all patients during first inpatient treatment prior to first surgical procedure, i.e., frame-based stereotactic biopsy or surgical tumor resection. In case of stereotactic biopsy and in contrast-enhancing tumors, the enhancing foci were targeted according to a standardized protocoll^13,14^. If applicable, areas with increased ^18^F]FET PET-uptake were targeted. Last follow-up was December 2022. Reference points of progression-free survival (PFS) and overall survival (OS) were set as the date of first surgical intervention to progression according to updated RANO criteria and date of death, respectively^15,16^. The datasets used and/or analysed during the current study available from the corresponding author on reasonable request.

### Imaging (MRI and [18F]FET PET)

Patients underwent MRI and [^18^F]FET PET scans within two weeks before invasive diagnostics by resection or biopsy. MRI and [^18^F]FET PET had to be obtained with 7 days difference or less. T1 sequences with and without contrast enhancement as well as T2 sequences were obtained in all patients. FLAIR sequences were not obtained in all patients and analysed if available. MRI scans were performed on 1.5 and 3.0 Tesla scanners (Philips Intera 3T, Andover, MA and Signa HDxt, GE Healthcare, Milwaukee, WI, USA). Gadolinium-based contrast agent (Gadopentetatedimeglumin, Magnevist, Schering, Berlin, Germany) was applied intravenously at a dose of 0.1 mmol/kg body weight. Tumor volumes in T2-weighted MRI scans was assessed through manual segmentation (HERMES Workstation, HERMES Medical Solutions, Stockholm, Sweden) by a faculty neurosurgeon specializing in glioma surgery. Furthermore, in cases where differentiation between edema and tumor tissue was difficult, the senior investigator (B.S.) was consulted and made the final decision. For differentiation between edema-related T2 alterations and putative nCEV, criteria described by *Lasocki et al.* were used^17^. Accordingly, edema typically spares grey matter, e.g., cortex and nuclei, while nCEV potentially invades grey matter. Edema spreads concentrically along anatomical structures and respects their borders while nCEV inflates and distorts brain parenchyma, oftentimes eccentrically. The hyperintensity of edema might be more pronounced and fades towards the periphery, whereas nCEV is characterized by only mild T2/FLAIR hyperintensity. The mass effect of edema might be less pronounced and, if large in size, be generalized. In comparison, T2 hyperintensity caused by nCEV oftentimes presents with a focal mass effect and parenchyma expansion (Suppl. Table 1). Dynamic [^18^F]FET PET scans were acquired utilizing Siemens ECAT EXACT HR + scanner (Siemens Healthineers, Erlangen, Germany). Fasting for six hours prior to image acquisition was mandatory. Approximately 180 MBq of [^18^F]FET PET was administered intravenously. Biological tumor volumes were measured by automated segmentation using a threshold for determination of [^18^F]FET PET-positivity of 1.6 tumor-to-brain ratio (TBR)^10,18–21^. The overlap between nCEV and BTV was determined by automated fusion of the two modalities after transferral to the HERMES workstation and using HERMES Hybrid Viewer 2.5.

### Relative anatomical and biological tumor volumes

**Fig. 1:**
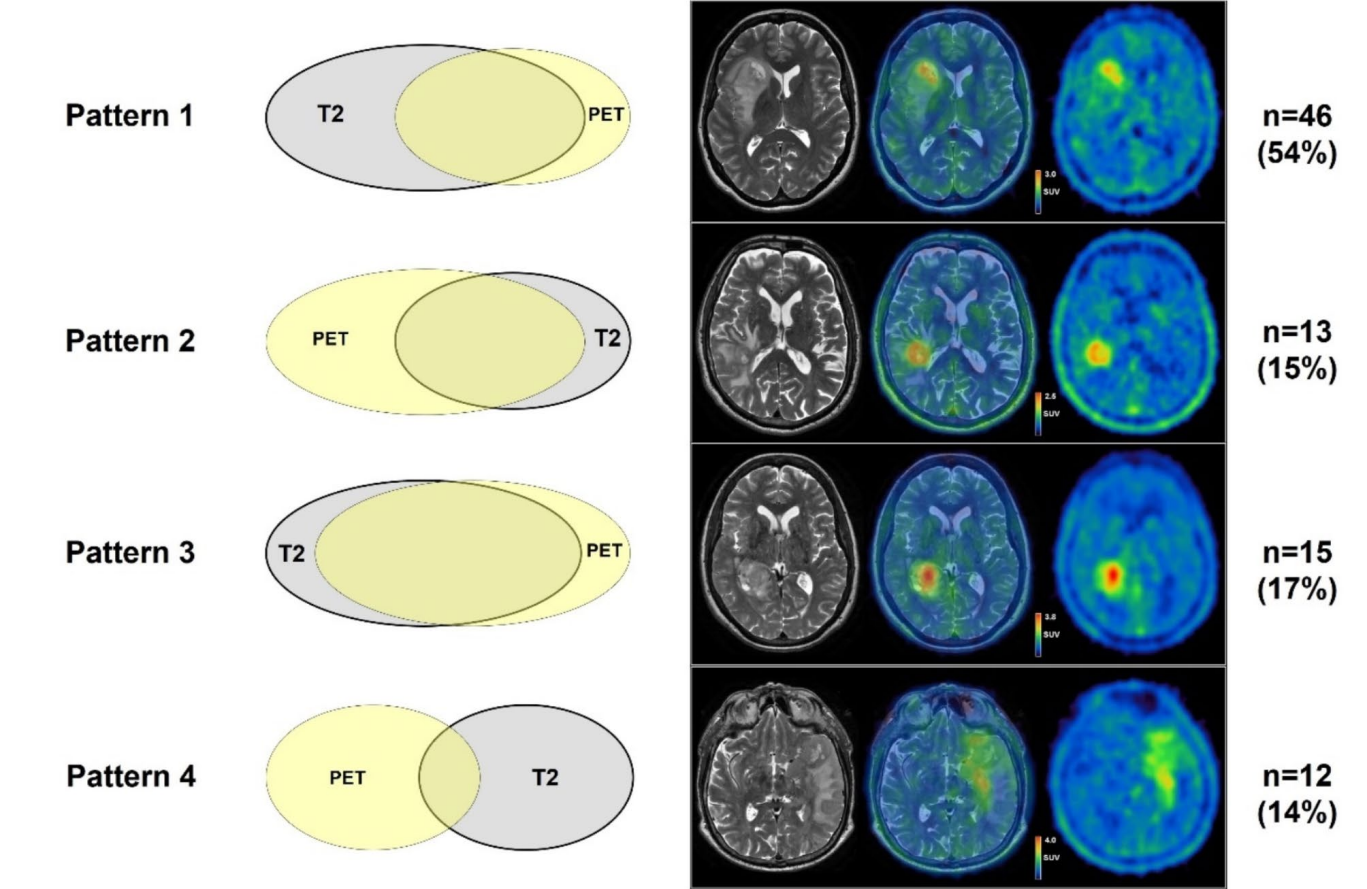
Schematic illustration and examples of different volume patterns in juxtaposition. Different patterns were defined based on the extent of overlap between non-enhancing tumor volume and biological tumor volume as determined by volumetric analysis of [^18^F]FET PET imaging. The number of patients in each group and percentages are given. T2 = non-enhancing tumor volume in T2-weighted MRI-scan; PET = biological tumor volume in [^18^F]FET PET.

Based on the extent of overlap between nCEV and BTV, four different patterns were defined (Fig. 1). The term ‘intersectional tumor volume’ is hereby defined as the overlapping volume between nCEV and BTV as assessed by [^18^F]FET PET. The proportion of intersectional tumor volume to nCEV was defined as ‘x’. Conversely, the proportion of intersectional tumor volume to BTV was defined as ‘y’.

$$\text{x} = \:\frac{intersectional\:tumor\:volume}{nCEV}, \,\,\text{y} = \:\frac{intersectional\:tumor\:volume}{BTV}$$

Mathematically, patterns were defined as follows:

Pattern 1, nCEV is twice as large as the BTV and encloses all or most of it:

$$\:\frac{x}{y} < 0.5$$

Pattern 2, BTV is twice as large as the nCEV and encloses all or most of it:

$$\:\frac{x}{y} > 2.0$$

Pattern 3, there is no pronounced predominance of either volume; the intersectional volume is at least half the size of the respective exclusive volumes:

$$0.5 < \:\frac{x}{y} < 2.0 \,\,and\,\, \text{x} > 0.5; \text{y} > 0.5$$

Pattern 4, there is no pronounced predominance of either volume; the intersectional volume is less than half the size of the respective exclusive volumes:

$$0.5 < \:\frac{x}{y} < 2.0 \,\,and\,\, \text{x} < 0.5; \text{y} < 0.5$$

### Histopathology and molecular markers

Histological diagnosis was established at the Center for Neuropathology of the LMU University Hospital Munich according to the CNS WHO 2007 classification initially and retrospectively reevaluated in accordance with the CNS WHO 2021 classification^22,23^. O6-methylguanine-DNA methyltransferase (MGMT) promoter methylation status was determined through methylation-specific PCR^24,25^. IDH mutational status was assessed using pyrosequencing of an 88-bp-long fragment for the *IDH1* gene with codon 132 analysis and an 83-bp-long fragment for the *IDH2* gene with codon 172 analysis. Microsatellite markers were used to detect allelic loss of chromosomes 1p and 19q.

### Treatment

Treatment decisions were made based on interdisciplinary tumor board recommendations as well as in accordance with patients’ preferences. Tumor-specific therapy comprised surgical glioma resection if safely feasible, radiotherapy and alkylating agent chemotherapy^26^. Patients with glioblastoma were treated with radiochemotherapy according to the Stupp protocol after histological sampling^27^.

### Statistics

Statistical analyses were performed utilizing SPSS 22.0 (SPSS, inc., Chicago, Illinois, USA) and GraphPad PRISM 9.4.1 (GraphPad software, inc., California, USA) software. Progression free survival (PFS) and overall survival (OS) were investigated through Kaplan-Meier estimator. For comparison of categorical, demographic data, chi-squared test and Fisher’s exact test were used if applicable. D’Agostino-Pearson test was used to investigate normal distribution. Parametric data was compared by means of Student’s t-tests and ANOVA. Non-parametric data was compared by Mann-Whitney U-test and Kruskal-Wallis tests. Uni- and multivariate analyses were performed through Cox proportional hazards regression models. Log-rank tests and Gehan-Breslow-Wilcoxon tests, a test that gives more weight to deaths at early time points, were computed. Values for Gehan-Breslow-Wilcoxon tests were only given in addition and were specifically outlined. The values given in the figures are based on Log-rank tests. P-values equal to or below 0.05 were termed significant.

## Results

86 patients with newly diagnosed glioma between 2007 and 2009 were included in this study. All patients had undergone preoperative MRI and [^18^F]FET PET imaging. Median age at diagnosis was 60 years (range 25–79 years) (Table 1). Diagnosis was IDHwt glioblastoma CNS WHO grade 4 in 70 patients (81%); IDH-mutant (IDHmut) astrocytoma CNS WHO grade 4 in 3 patients (4%), IDHmut astrocytoma CNS WHO grade 3 in 3 patients (4%), IDHmut astrocytoma CNS WHO grade 2 in 4 patients (5%) and oligodendroglioma CNS WHO grade 2 with 1p/19q-codeletion in 6 patients (7%). Ten patients (12%) showed no contrast enhancement on initial MRI. MGMT promoter methylation was seen in 44 patients (51%). If safely feasible, tumor resection was conducted prior to radiation or chemotherapy. 50 patients (58%) received stereotactic brain biopsy and 36 patients (42%) underwent tumor resection. Median follow-up time of surviving patients was 8.1 years, and only 14 patients (16%) were alive at database closure. 10 of 14 patients alive at data base closure had a IDHmut glioma. Median follow-up of the surviving patients was 97 months.

**Table 1:** Patient characteristics.

Factor	Number
Patients	86
Gender (f/m)	36/50
Age, y (median, range)	60, 25–79
KPS (median, range)	80, 40–100
CNS WHO grade (n with grade 4: n with grade 3: n with grade 2)	73:9:4
IDH mutational status (n with IDH mutation: n with IDH wildtype)	16:70
Surgical procedure	
Biopsy	50
Tumor resection	36

*KPS* Karnofsky performance status, *CNS* central nervous system, *WHO* World Health Organization, *IDH* isocitrate dehydrogenase.

## Intersectional volume patterns and outcome

Pattern 1, i.e., a larger nCEV encasing all or most of the smaller BTV, was most prevalent among the entire cohort and seen in 46 patients (54%). Patterns 2, 3 and 4 were similarly distributed and detected in 12 (14%), 15 (17%) and 13 (15%) patients respectively (Fig. 1). Within the glioblastoma cohort comprising 70 patients, 33 patients (47%) displayed pattern 1. Patterns 2, 3 and 4 were seen in 12 (17%), 14 (20%) and 11 (16%) patients (Fig. 1). In patients with IDHmut gliomas, 13 (81%) were diagnosed with pattern 1. Patterns 2, 3 and 4 were seen in one patient per pattern (6%). Thus, pattern 1 was observed at a significantly higher rate in patients with IDHmut gliomas (patients with glioblastoma vs. patients with IDHmut glioma displaying pattern 1, 47% vs. 81%, *p* = 0.02).

**Fig. 2:**
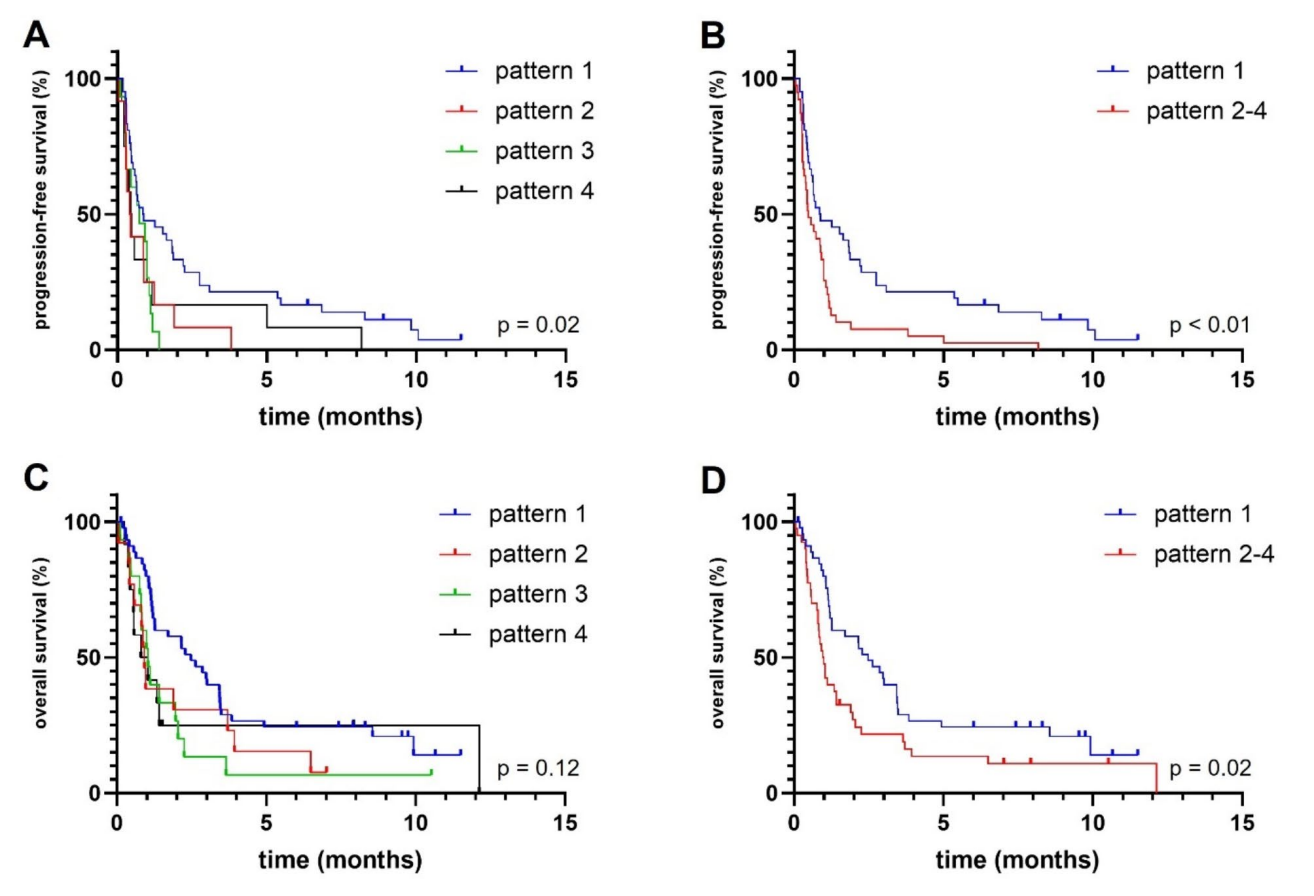
Progression-free survival (PFS) (**A**,**B**) and overall survival (OS) (**C**,** D**) of different intersectional tumor volume patterns. Pattern 1 was associated with significantly longer PFS than other patterns individually and combined ((**A**,**B**); *p* = 0.02 and *p* < 0.01). Pattern 1 was associated with longer OS than other patterns combined ((**D**), *p* = 0.02). Pattern 1: non-enhancing tumor volume (nCEV) is larger than the biological tumor volume (BTV) and encloses all or most of it. Pattern 2: BTV is larger than nCEV and encloses all or most of it. Pattern 3: nCEV and BTV are similar in size and there are minimal exclusive tumor volumes. Pattern 4: nCEV and BTV are similar in size, the exclusive tumor volumes are larger than the intersectional tumor volume.

**Fig. 3:**
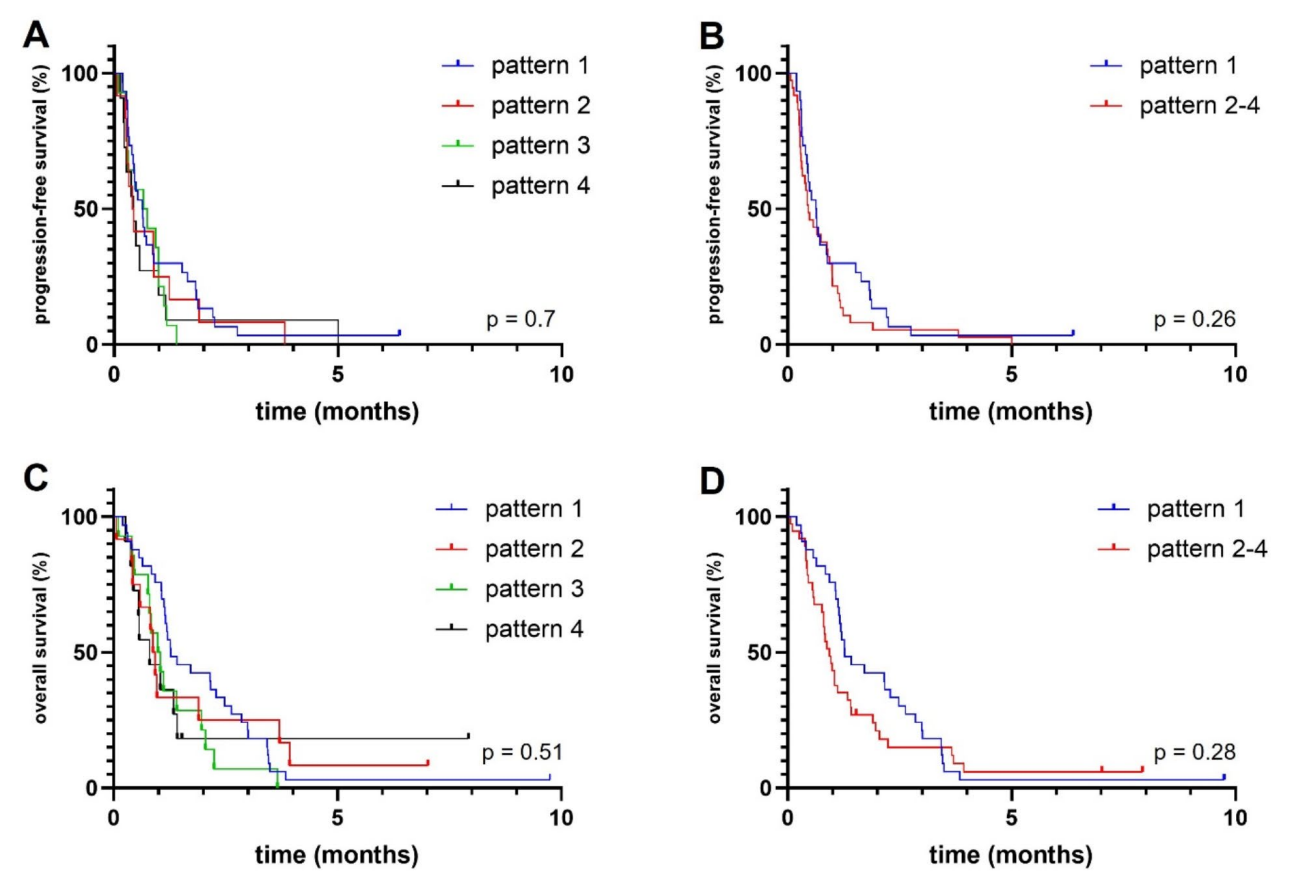
Progression-free survival (PFS) (**A**,**B**) and overall survival (OS) (**C**,**D**) of different intersectional tumor volume patterns among patients with newly diagnosed glioblastoma. There were no significant differences between the patterns in the glioblastoma cohort. Pattern 1: non-enhancing tumor volume (nCEV) is larger than the biological tumor volume (BTV) and encloses all or most of it. Pattern 2: BTV is larger than nCEV and encloses all or most of it. Pattern 3: nCEV and BTV are similar in size and there are minimal exclusive tumor volumes. Pattern 4: nCEV and BTV are similar in size, the exclusive tumor volumes are larger than the intersectional tumor volume.

In the entire cohort, median PFS was 10.3 (range: 6.5–14.0) and median OS was 16.9 (range 8.3–25.6) months. PFS and OS were longest in patients displaying pattern 1 with 10.4 and 29.8 months (Fig. 2). Shortest OS and PFS were seen in patients with pattern 2 (PFS, in months, 5.0; OS, in months, 11.0) and pattern 4 (PFS, in months, 5.5; OS, in months, 11.0) (Fig. 2). These patterns were both defined by larger portions of the BTV being spatially separated from the nCEV. Comparing pattern 1 (*n* = 46) with pattern 2 and 4 (*n* = 25), i.e., the two patterns with larger exclusive BTVs, PFS was significantly longer in patients displaying pattern 1 (PFS, in months; 10.4 versus 5.3, *p* = 0.01) and there was a trend towards longer OS (OS, in months; 29.8 versus 11, *p* = 0.08) (Suppl. Fig. 1 ). When looking exclusively at the glioblastoma cohort, there was no significant difference between the cohorts for PFS (*p* = 0.26) or OS (*p* = 0.28) with Log-rank (Mantel-Cox) test, but the number of patients with patterns other than pattern 1 were low (Fig. 3). When performing Gehan-Breslow-Wilcoxon test, survival was significantly longer in patients with pattern 1 (*p* = 0.04).

### Univariate and multivariate analysis

In the entire cohort, established molecular and clinical parameters such as age, methylated MGMT promoter, presence of an IDH mutation, lower CNS WHO grade and delay of therapy along with the intersectional volume pattern 1 were associated with a favorable PFS and OS times (Table 2). In the glioblastoma group, the outcome was comparable to the entire group (parameters such as CNS WHO grade, treatment and IDH status were not applicable here); the imaging parameter pattern 1 did not retain its significance for PFS or OS (Table 2). In multivariate analyses of the entire cohort, younger age was associated with longer progression-free and overall survival times. Pattern 1 was associated with prolonged PFS, but not OS. Presence of an IDH mutation was associated with longer OS (Table 3).

**Table 2:** Univariate analyses for progression-free and overall survival.

Parameters	Overall glioma group				Glioblastoma group			
	PFS		OS		PFS		OS	
	*P*	HR (CI 95%)	*P*	HR (CI 95%)	*p*	HR (CI 95%)	*P*	HR (CI 95%)
CNS WHO (2–4)	< 0.01*	1.9 (1.3-3.0)	**< 0.01**	3.14 (1.9–6.14)	n.a			
MGMT status (methylated)	< 0.01*	0.31 (0.18–0.54)	**< 0.01**	0.29 (0.17–0.49)	0.02*	0.52 (0.29–0.9)	**< 0.01**	0.5 (0.3–0.85)
IDH mutation present	< 0.01*	0.25 (0.12–0.47)	**< 0.01**	0.13 (0.05–0.28)	n.a.			
Age*	< 0.01*	1.04 (1.02–1.06)	**< 0.01**	1.04 (1.02–1.06)	0.11	1.02 (1.0-1.05)	**0.01**	1.03 (1.01–1.05)
Resection versus biopsy	0.28	0.44 (0.07–1.55)	0.94	0.98 (0.61–1.57)	0.48	0.47 (0.03–2.55)	0.21	0.72 (0.43–1.19)
Treatment^**1**^	0.01*	1.52 (1.12–2.14)	**< 0.01**	1.73 (1.26–2.48)	n.a.			
Pattern 1 versus other patterns	< 0.01*	0.48 (0.29–0.81)	**< 0.01**	0.53 (0.33–0.85)	0.19	0.7 (0.41–1.2)	0.16	0.7 (0.43–1.15)

*CNS* central nervous system, *WHO* World Health Organization, *MGMT* O-6-methylguanine-DNA methyltransferase, *IDH* isocitrate dehydrogenase. *Continuous variable; ^1^“wait-and-see”, “chemotherapy only”, “radiation only”, “radiochemotherapy”, *PFS* progression free survival, *OS* overall survival.

Significant values are in bold.

**Table 3:** Multivariate analyses for progression-free and overall survival.

Parameters	Overall glioma group			
	PFS		OS	
	*P*	HR (CI 95%)	*P*	HR (CI 95%)
CNS WHO (2–4)	0.84	1.08 (0.47–2.22)	0.74	1.19 (0.38–3.18)
IDH mutation present	0.12	0.36 (0.08–1.14)	0.05*	0.19 (0.03–0.8)
Age*	0.04*	1.02 (1.0-1.05)	0.03*	1.02 (1.0-1.05)
Treatment^1^	0.68	0.91 (0.6–1.43)	0.65	0.91 (0.61–1.41)
Pattern 1 versus other patterns	0.05*	0.59 (0.34-1.0)	0.1	0.66 (0.4–1.08)

*CNS* central nervous system, *WHO* World Health Organization, *IDH* isocitrate dehydrogenase. *Continuous variable; ^1^“wait-and-see”, “chemotherapy only”, “radiation only”, “radiochemotherapy”; *PFS* progression free survival, *OS* overall survival.

Significant values are in bold.

## Discussion

Anatomic tumor volumes measured by MRI can differ from BTVs as assessed by amino- acid PET in patients with newly diagnosed glioma. Several studies have previously shown that BTV is usually larger than the CEV, but smaller than the nCEV in primarily contrast enhancing tumor entities^7–9,12,28^. Furthermore, besides mere differences in size, the overlap of the corresponding tumor volumes is often low^12^. This phenomenon and its clinical implications have not been investigated in detail yet. We aimed at defining different intersectional volume patterns and report highly variable intersectional volume patterns among gliomas and poor prognosis of patients with BTVs that were spatially dissociated from the nCEV.

In this study, 86 patients with newly diagnosed glioma CNS WHO grades 2–4 were assigned to four different groups according to the pre-interventional overlap pattern between nCEV (as defined by T2 tumor volume) and BTV (Table 1). We found heterogenous spatial distributions of nCEVs and BTVs both in patients with glioblastoma and patients with IDHmut glioma. Most patients (54%) showed a larger nCEV than BTV and enclosing all or most of the BTV, termed pattern 1 (Fig. 1). This group showed the best prognosis. Patterns characterized by larger BTV and small intersectional, i.e., spatially dissociated, tumor volumes showed shortest PFS and OS times (Fig. 2). In multivariate analyses, pattern 1 and younger age were associated with longer PFS, potentially hinting towards a therapeutic effect of targeting BTV and T2 tumor volume with regards to surgery and radiotherapy planning as opposed to a potential undertreatment of patients displaying other patterns.

Patients with IDHmut glioma displayed pattern 1 significantly more often than patients with glioblastoma (81% versus 47%). Patient- and glioma derived prognostic factors did not differ from findings reported in large, prospective, clinical studies. Multivariate analyses showed a significant survival benefit in patients with younger age at diagnosis and IDHmut gliomas.

The relevance of nCEV for prognostication and therefore also treatment planning including resection and radiotherapy was recently unraveled in several studies. Especially in IDHmut malignant glioma, both resection as well as irradiation of the nCEV tumor portion have shown to be associated with improved outcome^29–33^. It has to be assumed that the nCEV harbors a significant portion of active tumor cells that might be responsible for a tumor recurrence^28^. This is in line with previous findings showing that glioblastoma recurrence often occurs within pre-irradiated parenchyma or close to the original tumor margin. While the incorporation of nCEV tumor part into irradiation planning might improve outcome, a too large resulting field of irradiation might be associated with a higher treatment related toxicity. In consequence, modulation of radiation treatment planning according to multiple imaging parameters, including PET derived BTV, might improve both tumor control and prolong tumor relapse^34^. In addition, BTV has been shown to harbor prognostic information and large pre-therapeutic BTVs have already been reported to be associated with poor prognosis^9^. However, current treatment plans oftentimes neglect amino-acid PET-derived BTV, although not taking BTV into account when planning radiotherapy might be a risk factor for out of field recurrence. The clinical implication of targeting BTV to prevent undertreatment has not yet been determined prospectively. Efforts are currently made to investigate the role of BTV irradiation in recurrent glioblastoma in a clinical phase II trial (NCT01252459).

Another interesting question is whether the different tumor volume intersection patterns might represent differences in growth/invasiveness characteristics. Previous data suggested that different volume patterns might to be associated with grading: Arbizu et al. reported divergent volume patterns in CNS WHO grade 3 and 4 gliomas^12^. Their data suggested a predominant pattern of BTV enclosed in larger MRI-volume in anaplastic astrocytomas while an opposite pattern was noted in glioblastoma. Our study did not investigate this hypothesis, however, our data show that different patterns can be found within one glioma entity. Further analyses are needed to investigate predominant, entity-specific intersectional volume patterns.

A limitation of this study might be a certain uncertainty concerning the delineation of nCEV as opposed to perifocal edema on T2/FLAIR-weighted images. Despite applying criteria by *Lasocki et al.*, certain differentiation is not yet possible on conventional imaging^17^. However, the inter-rater agreement for non-enhancing glioblastoma tissue on preoperative MRI is good and can be improved by relying on experts for tumor volume measurements^35^. This study is further limited by the low number of patients with IDHmut gliomas and the high probability of intercorrelation between variables. MGMT status, age and treatment were significant factors for outcome and all might be dependent on IDH mutational status. This renders multivariate analyses difficult to interpret. While BTVs were utilized for surgery planning in all patients and our center is a tertiary referral hospital, many patients received radiotherapy elsewhere and sufficient data on how often BTVs were utilized for radiotherapy target delineation could not be gathered. Accordingly, our data does not allow for a conclusion on the benefit of targeting BTV additionally. This question might be answered by the GLIAA-study (NCT01252459).

In summary, we report a high variability in intersectional volume patterns in glioma. Most frequently, the BTV is encased by the nCEV, but other patterns are not rare. Tumors that display a volume intersection pattern with considerable BTV portion outside the corresponding nCEV might show shorter progression-free survival. This might reflect a potential negative association between a mere MRI sequences-based surgery and radiotherapy target volume planning resulting in a possible undertreatment of metabolically active glioma tissue. Clinical trials evaluating inclusion of PET-derived volumes in treatment planning are urgently needed and in part already ongoing.

## Electronic supplementary material

Below is the link to the electronic supplementary material.

Supplementary Material 1

## Data Availability

The datasets used and/or analysed during the current study available from the corresponding author on reasonable request.
