# Supplementary material for: Patterns of intersectional tumor volumes in T2-weighted MRI and [18F]FET PET in adult glioma: a prospective, observational study
Source: Sci Rep. 2024 Oct 4;14:23071. doi: 10.1038/s41598-024-73681-5 (PMC11452397; doi:10.1038/s41598-024-73681-5)
Supplement: Supplementary file 1 — Supplementary Material 1 [file 41598_2024_73681_MOESM1_ESM.pdf]

Supplementary material

Suppl. Table 1. Differentiation between edema and non-contrast-enhancing tumor

**Differential features on T2/FLAIR-weighted MRI**	
**Edema**	**nCEV**
Marked hyperintensity, peripheral fading	Mild hyperintensity
Concentric, finger-like spread	Eccentric spread
Grey matter sparing	Grey matter infiltration and inflation
Respecting anatomical borders	Distortion of anatomical structures
Mass effect if generalized	Focal mass effect

Adapted by *Lasocki A et al., AJNR Am J Neuroradiol., 2019*

FLAIR, Fluid attenuated inversion recovery; MRI, magnetic resonance imaging; nCEV, non-contrast-enhancing tumor volume.

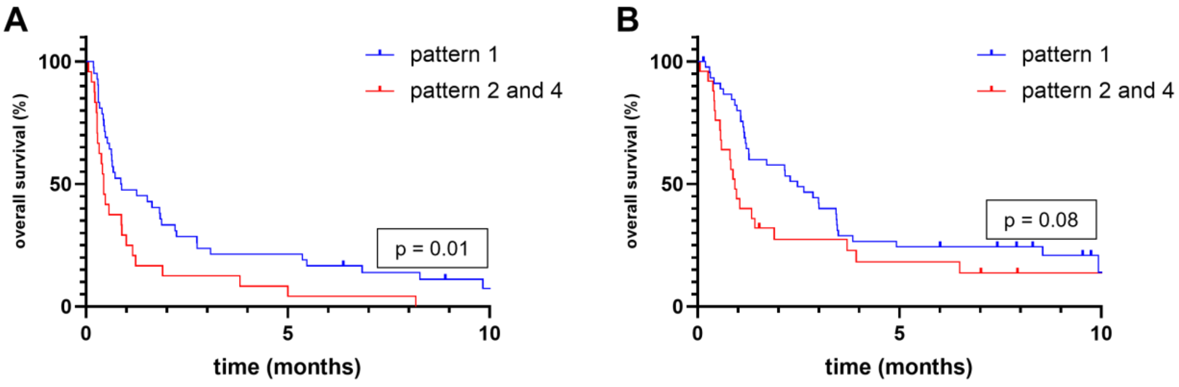

Suppl. Fig. 1. Progression-free survival (PFS) (A) and overall survival (OS) (C) of intersectional tumor volume pattern 1 versus pattern 2 and 4. Pattern 1 was associated with significantly longer PFS than patterns 2 and 4 (PFS, in months; 10.4 versus 5.3, p=0.01) (A). There was a trend towards longer OS in patients with pattern 1 (OS, in months; 29.8 versus 11, p=0.08). Pattern 1: non-enhancing tumor volume (nCEV) is larger than the biological tumor volume (BTV) and encloses all or most of it. Pattern 2: BTV is larger than nCEV and encloses all or most of it. Pattern 4: nCEV and BTV are similar in size, the exclusive tumor volumes are larger than the intersectional tumor volume.
